# Variation in the link between oxygen consumption and ATP production, and its relevance for animal performance

**DOI:** 10.1098/rspb.2015.1028

**Published:** 2015-08-07

**Authors:** Karine Salin, Sonya K. Auer, Benjamin Rey, Colin Selman, Neil B. Metcalfe

**Affiliations:** 1 Institute of Biodiversity, Animal Health and Comparative Medicine, College of Medical, Veterinary and Life Sciences, University of Glasgow, Glasgow, UK; 2 Laboratoire de Biométrie et Biologie Évolutive, UMR 5558, CNRS, Université de Lyon 1, Lyon, France; 3 Brain Function Research Group, School of Physiology, Faculty of Health Sciences, University of the Witwatersrand, Johannesburg, South Africa

**Keywords:** mitochondrial coupling efficiency, life history, oxidative stress, reactive oxygen species, trade-off, uncoupling

## Abstract

It is often assumed that an animal's metabolic rate can be estimated through measuring the whole-organism oxygen consumption rate. However, oxygen consumption alone is unlikely to be a sufficient marker of energy metabolism in many situations. This is due to the inherent variability in the link between oxidation and phosphorylation; that is, the amount of adenosine triphosphate (ATP) generated per molecule of oxygen consumed by mitochondria (P/O ratio). In this article, we describe how the P/O ratio can vary within and among individuals, and in response to a number of environmental parameters, including diet and temperature. As the P/O ratio affects the efficiency of cellular energy production, its variability may have significant consequences for animal performance, such as growth rate and reproductive output. We explore the adaptive significance of such variability and hypothesize that while a reduction in the P/O ratio is energetically costly, it may be associated with advantages in terms of somatic maintenance through reduced production of reactive oxygen species. Finally, we discuss how considering variation in mitochondrial efficiency, together with whole-organism oxygen consumption, can permit a better understanding of the relationship between energy metabolism and life history for studies in evolutionary ecology.

## 1. Introduction

Scientists have long acknowledged the importance of estimating an animal's metabolic rate, given its potential impact on the rate of resource uptake from the environment and allocation of those resources [1–4]. Traditionally, investigators have tended to use whole-animal measures of oxygen consumption as a proxy for the rate of metabolism [1]. Studies across a broad range of taxa have shown that intraspecific variation in the rate of oxygen consumption is related to performance across a diverse range of traits including work output [5,6], growth rate [7], degree of aggressiveness [8], feeding rate [9], lactation capacity [10] and lifespan [11–13]. However, the association between oxygen consumption and performance is not straightforward, with studies reporting positive, negative or no relationship, depending on the species and trait investigated (reviewed in [14]). While these inconsistent findings are triggering extensive debates centred on theoretical and methodological issues [11,14–21], what is often overlooked is that whole-organism oxygen consumption may be only a partial proxy for energy metabolism.

The main issue here is that oxygen consumption itself, whether measured on a whole-organism or tissue-specific level, is not a true measure of adenosine triphosphate (ATP) production, since the amount of ATP generated per unit of oxygen consumed can vary significantly [22]. Evidence for this variability has become much more apparent recently with the dramatic increase in fundamental mitochondrial research, primarily in the fields of biochemistry and biomedicine [22,23]. This research has been driven, in part, by technological advances and methodological developments that have enabled determination of mitochondrial function with improved resolution, higher sensitivity and the requirement for less biological material (box 1). It is now evident that variation in the efficiency with which mitochondria produce ATP exists between individuals [5,31,32], populations [33,34] and environments [35], and even within the same individual over time [6]. This spatial and temporal variability in mitochondrial functioning adds an additional layer of understanding to our studies of metabolism. The existence of variation in the mitochondrial functioning means that findings using oxygen consumption as a measure of energy metabolism, while neither an inaccurate nor an inappropriate proxy of the rate of aerobic metabolism, requires careful interpretation with respect to the amount of ATP produced [36].

Box 1. **Practical aspects of mitochondrial assessment.**

Methods for measuring the P/O ratio in isolated mitochondria were developed more than 50 years ago by Chance & Williams [24]. Protocols have also been developed that allow the use of permeabilized cells [25] or homogenized tissue, which permit rapid sample preparation and give the highest yield of mitochondrial extraction [26] (but see [27]). These various *in vitro* approaches are capable of providing a relevant indication of *in vivo* mitochondrial functioning [28], and recent technological developments have made such measurements feasible for a broad taxonomic range of organisms. While oxygen sensors have existed for decades, only recently has their resolution become sensitive enough to allow measurement of mitochondrial functioning from small tissue samples (50–100 mg, depending on tissue type and species), and from species with a low mitochondrial density and activity [29,30].

The goal of this review is to emphasize the importance of considering mitochondrial function together with the rate of oxygen consumption when investigating the link between energy metabolism and animal performance. We will first discuss potential limitations of employing measures of whole-animal oxygen consumption by itself to investigate energy metabolism. Second, we present empirical evidence that environmental factors can induce variation in mitochondrial efficiency and that this natural variation can have significant consequences for animal performance. We then consider why natural selection has not actually maximized mitochondrial efficiency, centring our explanation on another aspect of mitochondria: the generation of reactive oxygen species (ROS). Finally, we identify potential research questions that we feel would benefit from combined measures of mitochondrial efficiency and whole-animal oxygen consumption rates. Currently, there are few examples of the integration of bioenergetics into the realms of ecology and evolutionary biology (but see [33–38]). Therefore, the primary aim of this review is to stimulate researchers to adopt a much broader bioenergetics perspective, and in so doing produce a more integrative approach to the study of metabolism in the context of ecology and evolutionary biology.

## 2. What the rate of oxygen consumption does and does not tell us

There are a number of different definitions of metabolism [14,20], but in almost all cases the metabolism is measured by quantifying the rate of oxygen consumption. However, what assessments of oxygen consumption are expected to reveal with regard to energy metabolism is rarely specified. The broad concept of metabolism includes all anabolic and catabolic reactions within an organism, and consequently covers all processes associated with obtaining, assimilating, transforming and allocating resources. The term ‘oxygen consumption rate’ is usually presumed to be related to any, if not all, of these processes.

Energy derived from nutrients (carbohydrates, lipids, proteins) becomes usable only after being transformed into high-energy phosphate bonds in molecules of ATP. ATP is the principal energy source for most cellular functions, such as DNA, RNA and protein synthesis, cell division, signalling transduction pathways, muscle contractile activities, and active transport across the cell membrane [39]. The main sites of energy transformation are the mitochondria, which provide over 90% of cellular ATP [40]. The majority of ATP is produced via oxidative phosphorylation, a process through which substrate molecules are first oxidized by the tricarboxylic acid cycle to produce the reducing cofactors NADH and FADH_2_. These reducing agents pass electrons through a set of protein complexes (the electron transport chain—ETC) situated within the inner mitochondrial membrane and then to the final acceptor, oxygen. The electron flow through the ETC allows the proton (H^+^) pumps to expel H^+^ from the matrix to the mitochondrial inter-membrane space. The accumulation of H^+^ within this inter-membrane space generates an electrical (Δ*Ψ*_m_) and chemical (ΔpH) gradient, the electrochemical potential (Δp), that is required by the protein complex ATP synthase to drive the phosphorylation of ADP to ATP [41].

Although ATP production depends on the rate of oxidation, the number of ATP molecules produced for each oxygen atom consumed by the mitochondria (termed the P/O ratio) can vary [22]. One factor underlying this variation is that the amount of H^+^ pumped into the inter-membrane space per unit of oxygen consumed by the ETC (the H^+^/O ratio) is substrate-specific. For example, mitochondria oxidizing succinate exhibit an H^+^/O ratio of 6, whereas this ratio is 10 when the substrate is pyruvate/malate [22]. Despite well-established methods to determine which metabolic substrate is being oxidized (box 2), and direct evidence of variation in H^+^/O ratio due to substrate type [22,42], most studies of energy metabolism do not take into account this effect of substrate type on the efficiency of ATP production. A second potential mechanism altering the P/O ratio is slippage of the proton pumps. This slippage results in either fewer protons being pumped into the inter-membrane space per electron transferred by the ETC, or fewer ATP molecules produced per proton passing back through ATP synthase [22,43–45]. However, while such slip reactions can occur under certain conditions *in vitro*, their importance under physiological conditions remains uncertain [22].

Box 2. **Identifying the energy substrate used by the mitochondria.**

The nature of the metabolic substrate oxidized by the mitochondria affects not only the P/O ratio but also the ratio of carbon dioxide produced to oxygen consumed, also called the respiratory quotient (RQ) [39]. An advantage of the RQ is that it can be measured *in vivo*; it may be used to infer the relative contributions of carbohydrate and fat as substrates of the aerobic metabolism, where RQ = 1.0 indicates exclusively carbohydrate and RQ = 0.71 indicates exclusively fat [22,39]. All else being equal, the difference in P/O ratio between carbohydrate and fat oxidation leads to the calculation that, for a given oxygen consumption rate, the production of ATP should be 15% lower when oxidizing carbohydrate compared with fat [42]. Intermediate values are more difficult to interpret since these imply the utilization of combinations of substrates, including protein in some cases; the use of protein can be evaluated by measuring rates of nitrogen excretion, so potentially allowing calculation of the relative use of fat and carbohydrate substrates [39].

A third cause of variation in the P/O ratio, which has received much more attention (so will be the major focus of this article), is caused by a dissipation of Δp across the inner mitochondrial membrane. The dissipation of Δp is associated with an increase in heat production [46]. Several distinct biological mechanisms can alter Δp and hence affect the P/O ratio. The composition of the mitochondrial inner membrane, both in terms of its phospholipid fatty acids and the occurrence and activity of mitochondrial carrier proteins such as uncoupling proteins (UCP) or adenine nucleotide transporters (ANT), can influence the mitochondrial membrane conductance of protons and in turn the Δp [46–49]. The Δp is also likely to be affected by the active transport of cations (e.g. Ca^2+^), anions (e.g. ADP^3−^ and ATP^4−^) and metabolites (e.g. aspartate and glutamate) across the inner membrane [45,50]. Variation in P/O ratio has been discussed mostly in terms of proton leakage across the membrane, probably because it is the major contributor to the drop of Δp (and consequent increase in oxygen consumption) that is independent of ATP generation [43,51,52]. For instance, the futile cycle of H^+^ pumping and H^+^ leakage within rat liver and muscle mitochondria is estimated to account for approximately 20% of whole-animal oxygen consumption [53]. Thus, a significant proportion of the oxygen that an organism, a tissue or a mitochondrion consumes may not result in ATP production.

The degree of coupling of energy derived from oxidation to the generation of ATP varies across different tissues, individuals, environment and species, and over the lifetime of an individual [49,53–56], leading to variation in the efficiency of ATP production. Whole-organism oxygen consumption measures only the rate of substrate oxidation, and consequently does not distinguish between the energy used to produce ATP and energy dissipated through H^+^ leakage. Consequently, it cannot be used as an accurate proxy for ATP production when variation in the P/O ratio occurs. In this review, we examine this idea and discuss the importance of variation in P/O ratio in the context of evolutionary ecology. There has recently been an increase in empirical support for the concept that variation in the P/O ratio may be in response to ecological parameters (see §3) and have implications for animal performance (see §4). Two main approaches for estimating the P/O ratio are used, although other methods exist [50,57]. The first of these determines the mitochondrial oxygen consumption associated with the disappearance of a known amount of ADP (presumed converted to ATP). The second approach, developed by Lemasters [58], measures ATP production directly, so allowing calculation of the ATP : O ratio. These ratios, although not equal in terms of relevance and applicability, allow useful inferences to be made about the variation in P/O ratio.

## 3. Environmental causes of variation in the P/O ratio

There is clear evidence that environmental conditions can affect the efficiency of ATP production, potentially contributing to variability in performance over the lifetime of an individual. One of the most pervasive factors affecting the energy metabolism of organisms is the ambient temperature of the environment. Not surprisingly, the effect of temperature on mitochondria differs between poikilothermic ectotherms and endotherms. In poikilothermic ectotherms, increasing ambient temperature directly enhances the rate of biochemical reactions and thereby stimulates whole-organism oxygen consumption, but only up to a point beyond which reactions decrease as a result of damage. However, ambient temperature can also affect the efficiency of ATP production, presumably due to an increase in H^+^ conductance of the inner membrane with increasing temperature. This results in a greater proportion of H^+^ being shunted away from ATP synthase, thereby reducing the P/O ratio [59,60]. Temperature effects on the P/O ratio may therefore contribute to the non-proportional relationship between ATP production and the rate of oxygen consumption in poikilothermic ectotherms. The P/O ratio in endotherms is less directly affected by the ambient thermal regime, except when chronically exposed to cold temperatures [46,61,62]. In mammalian brown adipose tissue, prolonged cold exposure induces a severe reduction in mitochondrial coupling as a result of activation of uncoupling protein UCP1, and this mechanism clearly contributes to thermogenesis [46]. However, while acclimation to cold temperatures increases the rate of oxygen consumption in both mammals and birds, its effect on the efficiency of energy transduction appears to be taxon-specific: activation of UCPs has no effect on the coupling of ATP production to oxygen consumption in avian skeletal muscle [61]. The taxon-specific effect of cold acclimation on mitochondrial efficiency may be due to differences in the mechanism that is responsible for this acclimation, since, in birds, ANT rather than UCP is believed to be the principal mitochondrial uncoupler [62].

Food intake can also affect the efficiency of ATP production. Individuals with reduced food intake tend to exhibit an increase in the P/O ratio (e.g. by an average of 15% in fasting king penguins *Aptenodytes patagonicus* [35]). This is thought to be beneficial since an increase in P/O ratio minimizes the cost of ATP synthesis, thereby reducing energy substrate requirements [63]. Such plasticity may confer a physiological advantage by helping animals cope with periodic decreases in food intake [35]. It should be noted that this change in P/O ratio affects calculations of the metabolism of food-deprived animals, if these calculations are based on measures of oxygen consumption. For example, king penguins reduced their rate of oxygen consumption by 30% when fasting [35,64], but the reduction in ATP synthesis was much smaller than this would imply due to the associated 15% increase in P/O ratio [35].

Diet composition can also have an indirect effect on the P/O ratio (in addition to any direct effect of energy substrate on the ETC mentioned in §2), since it can affect the phospholipid properties of the inner mitochondrial membrane. For instance, the mitochondria of mice consuming a diet enriched in highly unsaturated fatty acids exhibited enhanced permeability to protons, greater mitochondrial proton leak and a decrease in the Δ*Ψ*_m_ [65], which would have altered the P/O ratio [66].

## 4. Consequences of the P/O ratio for animal performance

The P/O ratio can affect performance in a range of whole-organism traits (table 1). One of the most widely documented effects of the P/O ratio is on the rate of growth. The synthesis of new tissue is energetically costly, so investment in growth must depend on the capacity to generate ATP. The remarkable natural variation between individuals in their growth efficiency has been found to be related to mitochondrial functioning, largely as a result of research undertaken by animal production scientists investigating why some individuals grow faster than others on the same food ration [31]. Studies designed to explore the physiological basis for differences in growth efficiency in poultry show that individuals with higher growth efficiencies exhibit lower UCP expression in their muscle mitochondria [71,73] and reduced proton leak, resulting in a higher P/O ratio [71]. A similar relationship has also been reported in the common frog *Rana temporaria*, where individuals from natural populations characterized by larger average body size had a higher P/O ratio [33]. Surprisingly, the opposite trend was found in wild populations of garter snakes *Thamnophis elegans* [34].

**Table 1.** Examples of the relationship between mitochondrial coupling efficiency (P/O ratio: high values indicate that relatively little oxygen is required to produce a given amount of ATP) and animal performance indicators such as growth (**G**), reproduction (**R**) and somatic maintenance and lifespan (**M**) among conspecifics. ‘Experimental’ indicates whether the P/O ratio was manipulated (i.e. by use of uncoupling agents), so providing stronger evidence of a causal relationship than a simple correlation. ‘Assumed’ indicates that P/O was not measured but was assumed to have been decreased by use of an uncoupling agent.

species	experimental	tissue	P/O ratio	higher P/O ratio correlated with	refs
yeast (*Saccharomyces cerevisiae*)	yes		assumed	**M**: greater replicative lifespan; higher hydrogen peroxide production	[67]
fruitfly (*Drosophila melanogaster*)	yes		assumed	**M**: shorter lifespan; better viability when deprived of food	[68]
common frog tadpole (*Rana temporaria*)	yes	whole organism	ATP : O	**G**: higher growth rate **M**: greater hydrogen peroxide production; more lipid oxidative damage	[36]
common frog (*Rana temporaria*)	no	liver	ATP : O	**G**: larger adult body size	[33]
garter snake (*Thamnophis elegans*)	no	liver	ATP : O	**G**: smaller adult body size **R**: smaller neonate birth size; smaller litter size; earlier sexual maturation **M**: longer lifespan; more DNA oxidative damage (erythrocytes) when exposed to UV; greater oxidative repair efficiency; higher rate of trematode infection	[34]
broiler chicken (*Gallus gallus domesticus*)	no	duodenum	ADP : O	**G**: lower weight gain per food ingested **M**: less hydrogen peroxide production; less protein oxidative damage	[69,70]
broiler chicken (*Gallus gallus*)	no	skeletal muscle	ADP : O	**G**: higher growth rate	[71]
zebra finch (*Taeniopygia guttata*)	yes		assumed	**R**: greater egg production **M**: higher response to immune challenge	[37]
Swiss mice (*Mus musculus*)	yes		assumed	**G**: higher weight gain per food ingested; higher body mass **M**: shorter lifespan; more protein and DNA oxidative damage (brain, liver and heart)	[72]

The interpretation of results derived from correlative approaches is limited since any relationship between the P/O ratio and performance cannot be presumed causal. Recently, researchers have sought to manipulate the level of the P/O ratio in order to more directly test the effects of mitochondrial efficiency on whole animal traits [36,37,67,68]. An experimental decrease in the P/O ratio can be induced by chronically exposing the animal to an uncoupling agent such as 2,4-dinitrophenol (DNP), which acts, analogous to endogenous UCPs, to increase the rate of H^+^ translocation across the inner mitochondrial membrane. DNP exposure results in a decrease in the P/O ratio and a simultaneous increase in oxygen consumption [36,48,74]. Chronic exposure to DNP has revealed interesting effects of the P/O ratio on animal performance across a range of taxa (table 1). For example, rats *Rattus norvegicus* exposed to DNP had reduced exercise capacity [75], while mice exhibited a lower growth rate for a given food intake [72]. Common frogs exposed to DNP during the tadpole stage had a higher rate of oxygen consumption but grew more slowly than controls; they did not increase their food consumption and so were unable to compensate for the energy loss elicited by the decreased efficiency of ATP production [36]. However, it should be noted that DNP-treated zebra finches *Taeniopygia guttata* compensated for their mitochondrial inefficiency by increasing their food intake, and so maintained a body mass and rate of growth equivalent to that of controls [37].

Variation in the P/O ratio can also influence reproductive output. DNP-treated zebra finches laid fewer eggs, despite having a higher food consumption than controls [37]. By contrast, the artificial induction of mild uncoupling in yeast *Saccharomyces cerevisiae* led to an increased reproductive output, as a result of a greater replicative lifespan (see the discussion below as to why lower P/O ratio may increase lifespan [67]). However, there are presently too few studies to say definitively whether such divergent effects of uncoupling on performance are taxon-specific responses, or are due to differences in the degree of uncoupling, which is not always directly quantified and is rarely screened within a single study (but see [76]).

## 5. Why has selection not eradicated inefficient mitochondria?

Given the pervasive viewpoint that limited resources constrain individual investment across competing life-history traits [77], and that this resource limitation should apply as much to ATP as to the substrates used to produce it, one would expect a strong evolutionary selection pressure to maximize the P/O ratio in order to make the greatest use of the resources available. So why does variation in the P/O ratio persist? Perhaps the main reason for a submaximal P/O ratio is that ATP production must trade off against ROS generation. ROS are highly reactive molecules that have many beneficial properties, but also have the potential to induce cellular damage to lipids, proteins and nucleic acids if they escape neutralization by a suite of endogenous and exogenous antioxidants [78]. ROS are an inevitable consequence of the flow of electrons through the mitochondrial ETC [79], and their rate of generation is positively related to Δp [23,80]. Therefore, a decrease in Δ*Ψ*_m_ as a result of H^+^ leakage, for instance, while reducing the efficiency of ATP production, also decreases ROS production [23,81]. Experimental increases in H^+^ conductance (through DNP) can simultaneously reduce both the P/O ratio and oxidative damage [36,72] (but see also [37]). Since oxidative damage has been proposed as an important factor underlying both cellular and whole-organism senescence [78,82] (but see also [83]), a reduction in the P/O ratio could potentially be associated with a slower rate of ageing through its associated reduction in ROS production (the ‘uncoupling to survive’ hypothesis [23,84–86]). Lifespan has indeed been shown to be greater in individuals that have either a naturally lower Δp [13] or a higher H^+^ conductance as a result of exposure to uncoupling agents [67,68,72]. However, to date there is no direct *in vivo* evidence to show that a longer lifespan associated with a reduced P/O ratio is also associated with a reduction in ROS production.

The consequences of the P/O ratio for animal performance depend crucially on the extent to which changes in mitochondrial functioning affect the relative production of ATP versus ROS. Δp is the main mediator between the rate of respiration, ATP and ROS generation [41,45,80]. *In vitro* experiments show that when ATP synthesis is artificially inhibited, Δ*Ψ*_m_ increases (e.g. above 140 mV) and the rate of mitochondrial respiration drops to a low level; the consequent slowed rate of electron flow leads to the ETC becoming highly reduced, which promotes ROS production [45,87]. The low rate of mitochondrial respiration also leads to a build-up in the partial pressure of oxygen, which also contributes to an increase in ROS production. Conversely, an increase in ATP synthesis causes a drop in Δ*Ψ*_m_ (e.g. below 120 mV), thus accelerating the respiration rate and electron flow through the ETC [45,87]. This results in a decrease in the reduced state of the ETC and in oxygen partial pressure, both of which cause ROS production to drop to levels that are barely detectable [87] (but see also [88]). Thus, both ROS production and the efficiency of ATP production may be decreased by a slight drop in Δp [22,23]. However, the two phenomena occur at different Δp levels, and therefore the relationship between them is unlikely to be straightforward. Until recently, these processes could only be measured *in vitro*, resulting in an artificial alteration of Δp by modulating inhibitor and substrate supply. However, recent technological advances now allow researchers to simultaneously measure the rates of oxygen consumption and ATP production *in vivo* [28,57,86]. These studies have confirmed the *in vitro* finding that an experimental increase in H^+^ conductance results in lower ATP generation [74]. However, the recent development of more sophisticated and less cytotoxic uncoupling agents [89] and chemical probes to assess ROS generation *in vivo* [90] should help investigate the relationship between ATP and ROS generation.

## 6. Conclusion and directions for future research

We contend that the lack of appreciation for the variability in mitochondrial efficiency could lead to misleading interpretations of the relationships between oxygen consumption and animal performance, since the amount of ATP generated per molecule of oxygen consumed can vary significantly both among and within individuals. Combining sub-cellular and whole-organism measurements of metabolism will provide a more robust framework for understanding organismal energy metabolism. For example, a high P/O ratio does not necessarily result in high ATP production since this ratio can also be offset by a decrease in oxygen consumption rate (e.g. [36]); nor is it the case that individuals with a relatively low P/O ratio are necessarily producing less ATP than those with a higher P/O ratio, since this will depend on the rate of work of their mitochondria. Therefore, measuring both levels of energetic processes may give a better insight into the energy metabolism, since the rate of ATP generation is dependent on both the rate of oxygen consumption and the efficiency with which that consumed oxygen is used to make ATP.

The biological relevance of the P/O ratio for animal performance has often been evaluated using only a single tissue [33–35,37,61]. However, mitochondrial functioning, and in turn P/O ratio, may differ significantly among tissues in the same individual [56,91,92], probably due to tissue-specific control of the mitochondria [93,94]. For example, the effect of fasting on the P/O ratio of cold-acclimated birds differs between pectoralis and gastrocnemius muscles [95], and the loss of mitochondrial efficiency in older humans is more evident in dorsal interosseous muscle than in tibialis anterior muscle [86]. Thus, there is a need for studies that assess P/O ratio across multiple tissues. New approaches, based on biopsy [35,96] or blood samples [97], now make it possible for ecologists to conduct longitudinal studies of mitochondrial function [6], although such an approach may be challenging, depending on the tissue of interest or the studied animal. A crucial step in this field will be to assess whether samples that can be collected without sacrificing animals provide a representative measure of P/O ratios of different tissues.

Future research based on metabolic indices that integrate measurements of oxygen consumption rate and mitochondrial functioning may also aid interpretation of life-history variation among and within populations. While the concept of life-history trade-offs has been appreciated by evolutionary biologists for decades, the mechanisms underlying these trade-offs are still poorly understood [98]. If maximization of the P/O ratio is traded off against ROS production, among-individual differences in mitochondrial bioenergetics may play an important role in linking variation in resource allocation with oxidative stress, two components of a mechanism that has been proposed as a physiological basis of life-history trade-offs [23,99,100]. Ideally, studies of life-history trade-offs will need to consider and account for not only the rate and efficiency of energy metabolism (i.e. whole-organism oxygen consumption and mitochondrial efficiency respectively), but also for the consequent rate of ROS production.

In many situations, natural selection may favour phenotypes that have a relatively high efficiency of ATP production (since this may lead to faster growth, larger body size and/or greater reproductive output, as shown in table 1), but reducing oxidative stress may become a priority when self-maintenance takes precedence over growth or current reproductive rate [34,36,37]. While these two potential evolutionary trends seem to be mutually exclusive, the balance between maximizing mitochondrial energy efficiency versus operating at lowest oxidative cost may represent a population-, individual- or even stage-of-life-specific adaptive strategy [23,36,37]. For example, maximization of the P/O ratio might be expected during periods of high energy demand, such as during migration [101] or lactation [102], even if this results in greater ROS production that might, if uncontrolled, carry a cost that becomes evident later in life [103]. By contrast, in situations where food availability is high relative to energetic demands, then we might predict a greater prioritization of somatic maintenance relative to energy transduction efficiency, and hence a lower P/O ratio. As an example, this approach might help explain why individual Djungarian hamsters *Phodopus sungorus* on an *ad libitum* diet that spent a greater proportion of days in torpor (when energy demands are greatly reduced) also had slower rates of cellular senescence [104]. We might thus expect that this balance between the benefits of efficient ATP production and reduced ROS production may vary seasonally, in line with changing food availability and time windows for life-history events. The optimal P/O ratio and rate of oxygen consumption are likely to be shaped by both extrinsic (e.g. food availability, temperature) and intrinsic factors (such as genotype, substrate mobilization and hormonal state), but the existence of such a relationship between P/O ratio, oxygen consumption and life-history strategy still awaits experimental corroboration. Research in these areas should lead to the accumulation of sufficient information to allow a comprehensive meta-analysis of how the P/O ratio is affected by different environmental factors, and in turn how it influences life-history traits.

## Data accessibility

All data come from published articles.

## References

[1] Brown JH, Gillooly JF, Allen AP, Savage VM, West GB 2004 Toward a metabolic theory of ecology. Ecology 85, 1771–1789. (10.1890/03-9000)

[2] Rubner M 1908 Das problem der lebensdauer und seine beziehungen zu ernährung. Munich, Germany: R Oldenbourg.

[3] Pearl R 1928 The rate of living. New York, NY: Alfred A Knopf.

[4] Schmidt-Nielsen K 1984 Scaling, why is animal size so important? 1st edn Cambridge, UK: Cambridge University Press.

[5] Coen PM et al. 2012 Skeletal muscle mitochondrial energetics are associated with maximal aerobic capacity and walking speed in older adults. J. Gerontol. A Biol. Sci. Med. Sci. 68, 447–555. (10.1093/gerona/gls196) 23051977 PMC3593613

[6] Jacobs RA, Siebenmann C, Hug M, Toigo M, Meinild A-K, Lundby C 2012 Twenty-eight days at 3454-m altitude diminishes respiratory capacity but enhances efficiency in human skeletal muscle mitochondria. FASEB J. 26, 5192–5200. (10.1096/fj.12-218206) 22968913

[7] Auer SK, Salin K, Rudolf AM, Anderson GJ, Metcalfe NB 2015 The optimal combination of standard metabolic rate and aerobic scope for somatic growth depends on food availability. Funct. Ecol. 29, 479–486. (10.1111/1365-2435.12396)

[8] Biro PA, Stamps JA 2010 Do consistent individual differences in metabolic rate promote consistent individual differences in behavior? Trends Ecol. Evol. 25, 653–659. (10.1016/j.tree.2010.08.003) 20832898

[9] Gifford ME, Clay TA, Careau V 2014 Individual (co) variation in standard metabolic rate, feeding rate, and exploratory behavior in wild-caught semiaquatic salamanders. Physiol. Biochem. Zool. 87, 384–396. (10.1086/675974) 24769703

[10] Sadowska J, Gębczyński AK, Paszko K, Konarzewski M 2015 Milk output and composition in mice divergently selected for basal metabolic rate. J. Exp. Biol. 218, 249–254. (10.1242/jeb.111245) 25452500

[11] Speakman JR 2005 Body size, energy metabolism and lifespan. J. Exp. Biol. 208, 1717–1730. (10.1242/jeb.01556) 15855403

[12] Selman C, McLaren JS, Collins AR, Duthie GG, Speakman JR 2008 The impact of experimentally elevated energy expenditure on oxidative stress and lifespan in the short-tailed field vole *Microtus agrestis*. Proc. R. Soc. B 275, 1907–1916. (10.1098/rspb.2008.0355) PMC2596373 18467297

[13] Speakman JR et al. 2004 Uncoupled and surviving: individual mice with high metabolism have greater mitochondrial uncoupling and live longer. Aging Cell 3, 87–95. (10.1111/j.1474-9728.2004.00097.x) 15153176

[14] Burton T, Killen SS, Armstrong JD, Metcalfe NB 2011 What causes intraspecific variation in resting metabolic rate and what are its ecological consequences? Proc. R. Soc. B 278, 3465–3473. (10.1098/rspb.2011.1778) PMC3189380 21957133

[15] Burton T, Hoogenboom MO, Beevers ND, Armstrong JD, Metcalfe NB 2013 Among-sibling differences in the phenotypes of juvenile fish depend on their location within the egg mass and maternal dominance rank. Proc. R. Soc. B 280, 20122441 (10.1098/rspb.2012.2441) PMC3574412 23193132

[16] Clark TD, Sandblom E, Jutfelt F 2013 Aerobic scope measurements of fishes in an era of climate change: respirometry, relevance and recommendations. J. Exp. Biol. 216, 2771–2782. (10.1242/jeb.084251) 23842625

[17] Norin T, Malte H 2011 Repeatability of standard metabolic rate, active metabolic rate and aerobic scope in young brown trout during a period of moderate food availability. J. Exp. Biol. 214, 1668–1675. (10.1242/jeb.054205) 21525312

[18] Wone B, Sears MW, Labocha MK, Donovan ER, Hayes JP 2009 Genetic variances and covariances of aerobic metabolic rates in laboratory mice. Proc. R. Soc. B 276, 3695–3704. (10.1098/rspb.2009.0980) PMC2817310 19656796

[19] Careau V, Garland T 2012 Performance, personality, and energetics: correlation, causation, and mechanism. Physiol. Biochem. Zool. 85, 543–571. (10.1086/666970) 23099454

[20] Mathot KJ, Dingemanse NJ 2015 Energetics and behavior: unrequited needs and new directions. Trends Ecol. Evol. 30, 199–206. (10.1016/j.tree.2015.01.010) 25687159

[21] Glazier DS 2015 Is metabolic rate a universal ‘pacemaker’ for biological processes? Biol. Rev. 90, 377–407. (10.1111/brv.12115) 24863680

[22] Brand MD 2005 The efficiency and plasticity of mitochondrial energy transduction. Biochem. Soc. Trans. 33, 897–904. (10.1042/BST20050897) 16246006

[23] Brand MD 2000 Uncoupling to survive? The role of mitochondrial inefficiency in ageing. Exp. Gerontol. 35, 811–820. (10.1016/S0531-5565(00)00135-2) 11053672

[24] Chance B, Williams GR 1956 The respiratory chain and oxidative phosphorylation. Adv. Enzymol. Related Subjects Biochem. 17, 65–134. 10.1002/9780470122624.ch2 13313307

[25] Saks VA et al. 1998 Permeabilized cell and skinned fiber techniques in studies of mitochondrial function *in vivo*. Mol. Cell. Biochem. 184, 81–100. (10.1023/A:1006834912257) 9746314

[26] Pecinova A, Drahota Z, Nuskova H, Pecina P, Houstek J 2011 Evaluation of basic mitochondrial functions using rat tissue homogenates. Mitochondrion 11, 722–728. (10.1016/j.mito.2011.05.006) 21664301

[27] Larsen S, Kraunsoe R, Gram M, Gnaiger E, Helge JW, Dela F 2014 The best approach: homogenization or manual permeabilization of human skeletal muscle fibers for respirometry? Anal. Biochem. 446, 64–68. (10.1016/j.ab.2013.10.023) 24161612

[28] Ryan TE, Brophy P, Lin CT, Hickner RC, Neufer PD 2014 Assessment of *in vivo* skeletal muscle mitochondrial respiratory capacity in humans by near-infrared spectroscopy: a comparison with in situ measurements. J. Physiol. Lond. 592, 3231–3241. (10.1113/jphysiol.2014.274456) 24951618 PMC4146372

[29] Horan MP, Pichaud N, Ballard JWO 2012 Review: quantifying mitochondrial dysfunction in complex diseases of aging. J. Gerontol. Ser. A Biol. Sci. Med. Sci. 67, 1022–1035. (10.1093/gerona/glr263) 22459622

[30] Kuznetsov AV, Veksler V, Gellerich FN, Saks V, Margreiter R, Kunz WS 2008 Analysis of mitochondrial function in situ in permeabilized muscle fibers, tissues and cells. Nat. Protocols 3, 965–976. (10.1038/nprot.2008.61) 18536644

[31] Bottje WG, Carstens GE 2009 Association of mitochondrial function and feed efficiency in poultry and livestock species. J. Anim. Sci. 87, E48–E63. (10.2527/jas.2008-1379) 19028862

[32] van den Berg SAA et al. 2010 High levels of whole-body energy expenditure are associated with a lower coupling of skeletal muscle mitochondria in C57Bl/6 mice. Metabolism 59, 1612–1618. (10.1016/j.metabol.2010.03.008) 20494374

[33] Salin K, Roussel D, Rey B, Voituron Y 2012 David and Goliath: a mitochondrial coupling problem? J. Exp. Zool. A Ecol. Genet. Physiol. 317, 283–293. (10.1002/jez.1722) 25363578

[34] Robert KA, Bronikowski AM 2010 Evolution of senescence in nature: physiological evolution in populations of garter snake with divergent life histories. Am. Nat. 175, E147–E159. (10.1086/649595) 20050804

[35] Monternier P-A, Marmillot V, Rouanet J-L, Roussel D 2014 Mitochondrial phenotypic flexibility enhances energy savings during winter fast in king penguin chicks. J. Exp. Biol. 217, 2691–2697. (10.1242/jeb.104505) 24803465

[36] Salin K, Luquet E, Rey B, Roussel D, Voituron Y 2012 Alteration of mitochondrial efficiency affects oxidative balance, development and growth in frog (*Rana temporaria*) tadpoles. J. Exp. Biol. 215, 863–869. (10.1242/jeb.062745) 22323209

[37] Stier A, Bize P, Roussel D, Schull Q, Massemin S, Criscuolo F 2014 Mitochondrial uncoupling as a regulator of life-history trajectories in birds: an experimental study in the zebra finch. J. Exp. Biol. 217, 3579–3589. (10.1242/jeb.103945) 25063856

[38] Hill GE 2014 Cellular respiration: the nexus of stress, condition, and ornamentation. Integr. Comp. Biol. 54, 645–657. (10.1093/icb/icu029) 24791751

[39] Willmer P, Stone G, Johnston I 2009 Environmental physiology of animals. New York, NY: John Wiley & Sons.

[40] Lehninger AL, Nelson DL, Cosx MM 1993 Principles of biochemistry. New York, NY: Worrth Publishers.

[41] Mitchell P 1961 Coupling of phosphorylation to electron and hydrogen transfer by a chemi-osmotic type of mechanism. Nature 191, 144–148. (10.1038/191144a0) 13771349

[42] Welch KC, Altshuler DL, Suarez RK 2007 Oxygen consumption rates in hovering hummingbirds reflect substrate-dependent differences in P/O ratios: carbohydrate as a ‘premium fuel’. J. Exp. Biol. 210, 2146–2153. (10.1242/jeb.005389) 17562888

[43] Brown GC 1992 The leaks and slips of bioenergetic membranes. FASEB J. 6, 2961–2965. 1644259 10.1096/fasebj.6.11.1644259

[44] Groen BH, Berden JA, Vandam K 1990 Differentiation between leaks and slips in oxidative phosphorylation. Biochim. Biophys. Acta 1019, 121–127. (10.1016/0005-2728(90)90132-N) 2207111

[45] Kadenbach B 2003 Intrinsic and extrinsic uncoupling of oxidative phosphorylation. Biochim. Biophys. Acta Bioenerg. 1604, 77–94. (10.1016/S0005-2728(03)00027-6) 12765765

[46] Cannon B, Nedergaard J 2004 Brown adipose tissue: function and physiological significance. Physiol. Rev. 84, 277–359. (10.1152/physrev.00015.2003) 14715917

[47] Porter RK, Hulbert AJ, Brand MD 1996 Allometry of mitochondrial proton leak: influence of membrane surface area and fatty acid composition. Am. J. Physiol.-Regulat. Integr. Comp. Physiol. 271, R1550–R1560. 10.1152/ajpregu.1996.271.6.R1550 8997352

[48] Harper JA, Dickinson K, Brand MD 2001 Mitochondrial uncoupling as a target for drug development for the treatment of obesity. Obesity Rev. 2, 255–265. (10.1046/j.1467-789X.2001.00043.x) 12119996

[49] Brookes PS, Buckingham JA, Tenreiro AM, Hulbert AJ, Brand MD 1998 The proton permeability of the inner membrane of liver mitochondria from ectothermic and endothermic vertebrates and from obese rats: correlations with standard metabolic rate and phospholipid fatty acid composition. Comp. Biochem. Physiol. B Biochem. Mol. Biol. 119, 325–334. (10.1016/S0305-0491(97)00357-X) 9629666

[50] Hinkle PC 2005 P/O ratios of mitochondrial oxidative phosphorylation. Biochim. Biophys. Acta-Bioenerg. 1706, 1–11. (10.1016/j.bbabio.2004.09.004) 15620362

[51] Brand MD 1990 The proton leak across the mitochondrial inner membrane. Biochim. Biophys. Acta 1018, 128–133. (10.1016/0005-2728(90)90232-S) 2393654

[52] Porter RK, Brand MD 1993 Body mass dependence of H^+^ leak in mitochondria and its relevance to metabolic rate. Nature 362, 628–630. (10.1038/362628a0) 8385274

[53] Rolfe DFS, Brand MD 1997 The physiological significance of mitochondrial proton leak in animal cells and tissues. Biosci. Rep. 17, 9–16. (10.1023/A:1027327015957) 9171916

[54] Rolfe DFS, Brand MD 1996 Contribution of mitochondrial proton leak to skeletal muscle respiration and to standard metabolic rate. Am. J. Physiol.-Cell Physiol. 271, C1380–C1389. 10.1152/ajpcell.1996.271.4.C1380 8897845

[55] Hulbert AJ, Else PL, Manolis SC, Brand MD 2002 Proton leak in hepatocytes and liver mitochondria from archosaurs (crocodiles) and allometric relationships for ectotherms. J. Comp. Physiol. B Biochem. Syst. Environ. Physiol. 172, 387–397. (10.1007/s00360-002-0264-1) 12122455

[56] Hulbert AJ, Turner N, Hinde J, Else P, Guderley H 2006 How might you compare mitochondria from different tissues and different species? J. Comp. Physiol. B Biochem. Syst. Environ. Physiol. 176, 93–105. (10.1007/s00360-005-0025-z) 16408229

[57] Lanza IR, Nair KS 2010 Mitochondrial metabolic function assessed *in vivo* and *in vitro*. Curr. Opinion Clin. Nutr. Metab. Care 13, 511 (10.1097/MCO.0b013e32833cc93d) PMC3070485 20616711

[58] Lemasters JJ 1984 The ATP-to-oxygen stoichiometries of oxidative phosphorylation by rat liver mitochondria: an analysis of ADP-induced oxygen jumps by linear nonequilibrium thermodynamics. J. Biol. Chem. 259, 13 123–13 130. 6548475

[59] Brooks GA, Hittelman KJ, Faulkner JA, Beyer RE 1971 Temperature, skeletal muscle mitochondrial functions, and oxygen debt. Am. J. Physiol. 220, 1053–1059. 4323901 10.1152/ajplegacy.1971.220.4.1053

[60] Sommer AM, Portner HO 2004 Mitochondrial function in seasonal acclimatization versus latitudinal adaptation to cold in the lugworm *Arenicola marina* (L.). Physiol. Biochem. Zool. 77, 174–186. (10.1086/381468) 15095238

[61] Teulier L, Rouanet JL, Letexier D, Romestaing C, Belouze M, Rey B, Duchamp C, Roussel D 2010 Cold-acclimation-induced non-shivering thermogenesis in birds is associated with upregulation of avian UCP but not with innate uncoupling or altered ATP efficiency. J. Exp. Biol. 213, 2476–2482. (10.1242/jeb.043489) 20581277

[62] Walter I, Seebacher F 2009 Endothermy in birds: underlying molecular mechanisms. J. Exp. Biol. 212, 2328–2336. (10.1242/jeb.029009) 19617425

[63] Bevilacqua L, Ramsey JJ, Hagopian K, Weindruch R, Harper M-E 2005 Long-term caloric restriction increases UCP3 content but decreases proton leak and reactive oxygen species production in rat skeletal muscle mitochondria. Am. J. Physiol. Endocrinol. Metab. 289, E429–E438. (10.1152/ajpendo.00435.2004) 15886224

[64] Rey B, Halsey LG, Dolmazon V, Rouanet JL, Roussel D, Handrich Y, Butler PJ, Duchamp C 2008 Long-term fasting decreases mitochondrial avian UCP-mediated oxygen consumption in hypometabolic king penguins. Am. J. Physiol. Regulat. Integr. Compar. Physiol. 295, R92–R100. (10.1152/ajpregu.00271.2007) PMC2494815 18495832

[65] Stillwell W, Jenski LJ, Crump FT, Ehringer W 1997 Effect of docosahexaenoic acid on mouse mitochondrial membrane properties. Lipids 32, 497–506. (10.1007/s11745-997-0064-6) 9168456

[66] Yu L, Fink B, Herlein J, Oltman C, Lamping K, Sivitz W 2014 Dietary fat, fatty acid saturation and mitochondrial bioenergetics. J. Bioenerg. Biomembr. 46, 33–44. (10.1007/s10863-013-9530-z) 24121995 PMC3955408

[67] Barros MH, Bandy B, Tahara EB, Kowaltowski AJ 2004 Higher respiratory activity decreases mitochondrial reactive oxygen release and increases life span in *Saccharomyces cerevisiae*. J. Biol. Chem. 279, 49 883–49 888. (10.1074/jbc.M408918200) 15383542

[68] Padalko VI 2005 Uncoupler of oxidative phosphorylation prolongs the lifespan of *Drosophila*. Biochem. Moscow 70, 986–989. (10.1007/s10541-005-0213-1) 16266268

[69] Iqbal M, Pumford NR, Tang ZX, Lassiter K, Ojano-Dirain C, Wing T, Cooper M, Bottje W 2005 Compromised liver mitochondrial function and complex activity in low feed efficient broilers are associated with higher oxidative stress and differential protein expression. Poult. Sci. 84, 933–941. (10.1093/ps/84.6.933) 15971533

[70] Ojano-Dirain CP, Iqbal M, Cawthon D, Swonger S, Wing T, Cooper M, Bottje W 2004 Determination of mitochondrial function and site-specific defects in electron transport in duodenal mitochondria in broilers with low and high feed efficiency. Poult. Sci. 83, 1394–1403. (10.1093/ps/83.8.1394) 15339016

[71] Toyomizu M, Kikusato M, Kawabata Y, Azad MAK, Inui E, Amo T 2011 Meat-type chickens have a higher efficiency of mitochondrial oxidative phosphorylation than laying-type chickens. Comp. Biochem. Physiol. A Mol. Integr. Physiol. 159, 75–81. (10.1016/j.cbpa.2011.01.020) 21300168

[72] Caldeira da Silva CC, Cerqueira FM, Barbosa LF, Medeiros MH, Kowaltowski AJ 2008 Mild mitochondrial uncoupling in mice affects energy metabolism, redox balance and longevity. Aging Cell 7, 552–560. (10.1111/j.1474-9726.2008.00407.x) 18505478

[73] Tinsley N, Iqbal M, Pumford NR, Lassiter K, Ojano-Dirain C, Wing T, Bottje W 2010 Investigation of mitochondrial protein expression and oxidation in heart muscle in low and high feed efficient male broilers in a single genetic line. Poult. Sci. 89, 349–352. (10.3382/ps.2009-00138) 20075289

[74] Marcinek DJ, Schenkman KA, Ciesielski WA, Conley KE 2004 Mitochondrial coupling *in vivo* in mouse skeletal muscle. Am. J. Physiol. Cell Physiol. 286, C457–C463. (10.1152/ajpcell.00237.2003) 14522819

[75] Schlagowski AI, Singh F, Charles AL, Piquard F, Geny B, Zoll J 2013 Efficiency of skeletal muscle mitochondrial coupling is a critical factor for maximal exercise capacity and oxygen uptake in rats. Fundam. Clin. Pharmacol. 27, 33–33.

[76] Takahashi M, Sunaga M, Hirata-Koizumi M, Hirose A, Kamata E, Ema M 2009 Reproductive and developmental toxicity screening study of 2,4-dinitrophenol in rats. Environ. Toxicol. 24, 74–81. (10.1002/tox.20398) 18461559

[77] Stearns SC 1992 The evolution of life histories. Oxford, UK: Oxford University Press.

[78] Beckman KB, Ames BN 1998 The free radical theory of aging matures. Physiol. Rev. 78, 547–581. 9562038 10.1152/physrev.1998.78.2.547

[79] Murphy MP 2009 How mitochondria produce reactive oxygen species. Biochem. J. 417, 1–13. (10.1042/BJ20081386) 19061483 PMC2605959

[80] Korshunov SS, Skulachev VP, Starkov AA 1997 High protonic potential actuates a mechanism of production of reactive oxygen species in mitochondria. FEBS Lett. 416, 15–18. (10.1016/S0014-5793(97)01159-9) 9369223

[81] Miwa S, Brand MD 2003 Mitochondrial matrix reactive oxygen species production is very sensitive to mild uncoupling. Biochem. Soc. Trans. 31, 1300–1301. (10.1042/BST0311300) 14641047

[82] Harman D 1956 Aging—a theory based on free-radical and radiation-chemistry. J. Gerontol. 11, 298–300. (10.1093/geronj/11.3.298) 13332224

[83] Speakman JR, Selman C 2011 The free-radical damage theory: accumulating evidence against a simple link of oxidative stress to ageing and lifespan. Bioessays 33, 255–259. (10.1002/bies.201000132) 21290398

[84] Jastroch M, Keipert S, Meyer CW, Kutschke M, Heldmaier G, Klaus S, Oelkrug R 2010 Physiological significance of mitochondrial uncoupling protein 1 in the prevention of reactive oxygen species and control of substrate oxidation. FASEB J. 24, 1 (10.1096/fj.10-0101ufm) 20047894

[85] Keipert S, Voigt A, Klaus S 2011 Dietary effects on body composition, glucose metabolism, and longevity are modulated by skeletal muscle mitochondrial uncoupling in mice. Aging Cell 10, 122–136. (10.1111/j.1474-9726.2010.00648.x) 21070590 PMC3042149

[86] Amara CE, Shankland EG, Jubrias SA, Marcinek DJ, Kushmerick MJ, Conley KE 2007 Mild mitochondrial uncoupling impacts cellular aging in human muscles *in vivo*. Proc. Natl Acad. Sci. USA 104, 1057–1062. (10.1073/pnas.0610131104) 17215370 PMC1766336

[87] Barja G 2007 Mitochondrial oxygen consumption and reactive oxygen species production are independently modulated: implications for aging studies. Rejuvenation Res. 10, 215–224. (10.1089/rej.2006.0516) 17523876

[88] Cortassa S, O'Rourke B, Aon MA 2014 Redox-optimized ROS balance and the relationship between mitochondrial respiration and ROS. Biochim. Biophys. Acta Bioenerg. 1837, 287–295. (10.1016/j.bbabio.2013.11.007) PMC3902145 24269780

[89] Kenwood BM et al. 2014 Identification of a novel mitochondrial uncoupler that does not depolarize the plasma membrane. Mol. Metab. 3, 114–123. (10.1016/j.molmet.2013.11.005) 24634817 PMC3953706

[90] Cocheme HM et al. 2011 Measurement of H_2_O_2_ within living drosophila during aging using a ratiometric mass spectrometry probe targeted to the mitochondrial matrix. Cell Metab. 13, 340–350. (10.1016/j.cmet.2011.02.003) 21356523 PMC4413513

[91] Park S-Y et al. 2014 Cardiac, skeletal, and smooth muscle mitochondrial respiration: are all mitochondria created equal? AJP: Heart Circul. Physiol. 307, H346–H352. (10.1152/ajpheart.00227.2014) PMC4121645 24906913

[92] Salin K, Teulier L, Rey B, Rouanet JL, Voituron Y, Duchamp C, Roussel D 2010 Tissue variation of mitochondrial oxidative phosphorylation efficiency in cold-acclimated ducklings. Acta Biochim. Pol. 57, 409–412. 21125027

[93] Holmstrom MH, Iglesias-Gutierrez E, Zierath JR, Garcia-Roves PM 2012 Tissue-specific control of mitochondrial respiration in obesity-related insulin resistance and diabetes. Am. J. Physiol. Endocrinol. Metab. 302, E731–E739. (10.1152/ajpendo.00159.2011) 22252943

[94] Benard G, Bellance N, Jose C, Melser S, Nouette-Gaulain K, Rossignol R 2010 Multi-site control and regulation of mitochondrial energy production. Biochim. Biophys. Acta Bioenerg. 1797, 698–709. (10.1016/j.bbabio.2010.02.030) 20226160

[95] Monternier P-A, Fongy A, Hervant F, Drai J, Collin-Chavagnac D, Rouanet J-L, Roussel D In press. Skeletal muscle heterogeneity in fasting-induced mitochondrial oxidative phosphorylation flexibility in cold-acclimated ducklings. J. Exp. Biol. (10.1242/jeb.122671) 26026038

[96] Pesta D, Gnaiger E 2012 High-resolution respirometry: OXPHOS protocols for human cells and permeabilized fibers from small biopsies of human muscle. In Mitochondrial bioenergetics: methods and protocols (eds Palmeira CM, Moreno AJ), pp. 25–58. Totowa, NJ: Humana Press Inc. 10.1007/978-1-61779-382-0_3 22057559

[97] Stier A et al. 2013 Avian erythrocytes have functional mitochondria, opening novel perspectives for birds as animal models in the study of ageing. Front. Zool. 10, 33 (10.1186/1742-9994-10-33) 23758841 PMC3686644

[98] Zera AJ, Harshman LG 2001 The physiology of life history trade-offs in animals. Annu. Rev. Ecol. Syst. 32, 95–126. (10.1146/annurev.ecolsys.32.081501.114006)

[99] Monaghan P, Metcalfe NB, Torres R 2009 Oxidative stress as a mediator of life history trade-offs: mechanisms, measurements and interpretation. Ecol. Lett. 12, 75–92. (10.1111/j.1461-0248.2008.01258.x) 19016828

[100] De Block M, Stoks R 2008 Compensatory growth and oxidative stress in a damselfly. Proc. R. Soc. B 275, 781–785. (10.1098/rspb.2007.1515) PMC2596904 18182373

[101] Piersma T 2011 Why marathon migrants get away with high metabolic ceilings: towards an ecology of physiological restraint. J. Exp. Biol. 214, 295–302. (10.1242/jeb.046748) 21177949

[102] Speakman JR 2008 The physiological costs of reproduction in small mammals. Phil. Trans. R. Soc. B 363, 375–398. (10.1098/rstb.2007.2145) 17686735 PMC2606756

[103] Blount JD, Vitikainen EIK, Stott I, Cant MA In press. Oxidative shielding and the cost of reproduction. Biol. Rev. (10.1111/brv.12179) 25765468

[104] Turbill C, Smith S, Deimel C, Ruf T 2012 Daily torpor is associated with telomere length change over winter in Djungarian hamsters. Biol. Lett. 8, 304–307. (10.1098/rsbl.2011.0758) 21920955 PMC3297381

